# Allies or Enemies? The Power of Plant Hormones in Animals: Insights into Their Regulatory Roles

**DOI:** 10.3390/molecules30142984

**Published:** 2025-07-16

**Authors:** Agata Kućko, Agata Walczak, Emilia Wilmowicz, Bartłomiej Wolski, Katarzyna Wiktorska

**Affiliations:** 1 Department of Plant Physiology, Institute of Biology, Warsaw University of Life Sciences-SGGW, Nowoursynowska 159, 02-776 Warsaw, Poland; s205173@sggw.edu.pl; 2 Department of Plant Physiology and Biotechnology, Faculty of Biological and Veterinary Sciences, Nicolaus Copernicus University, 1 Lwowska Street, 87-100 Torun, Poland; emwil@umk.pl; 3 Department of Gynecology and Obstetrics, The Nicolaus Copernicus Hospital in Gdansk, Nowe Ogrody 1-6, 80-803 Gdansk, Poland; bwolski@copernicus.gda.pl; 4 Department of Physics and Biophysics, Institute of Biology, Warsaw University of Life Sciences-SGGW, Nowoursynowska 166, 02-787 Warsaw, Poland; katarzyna_wiktorska@sggw.edu.pl

**Keywords:** auxin, cancer, cytokinins, ethylene, jasmonates, phytohormones, plant hormones

## Abstract

Phytohormones, representing a diverse group of molecules, are essential in orchestrating plant growth and development, ensuring the smooth progression of the entire life cycle from germination to senescence. Emerging research reveals that these compounds also exert biological effects in non-plant systems, including animals. Although some phytohormones can be harmful, their health-promoting potential is rapidly gaining attention. This has sparked a growing interest in exploring plant hormones as novel therapeutic agents, particularly in precision medicine. This review brings together a multidisciplinary team—plant physiologists, a pharmacist, and a medical doctor—to delve into the latest insight surrounding the health-related impacts of plant hormones on animal systems, with a particular emphasis on human health. We comprehensively analyze their effects, weighing both the benefits and potential risks. Key phytohormones—auxin, abscisic acid, cytokinins, jasmonates, ethylene, strigolactones, and gibberellins—are highlighted for their remarkable regulatory roles in animal physiology, with a special focus on their implications for human health. Our discussion reveals how phytohormones may help address critical health challenges, particularly those related to aging populations, including neurodegenerative diseases, diabetes, and cancers. These plant-derived molecules are emerging as promising candidates for future drug development and nutritional therapies. Hence, a deeper understanding of phytohormone action may not just revolutionize agriculture but also open new frontiers in medicine and human health.

## 1. Introduction

Plant hormones (named phytohormones) are low-molecular-weight compounds synthesized in all plant tissues and species. In general, hormones are signaling molecules that are transported over long distances within the body to regulate various physiological functions. When a hormone binds to its receptor, it triggers a cascade of events known as the *signal transduction pathway*, which ultimately leads to modifications in the function of the target cell. Unlike animals, plants lack specialized glands; instead, every plant cell is capable of synthesizing hormones. A distinctive characteristic of phytohormones is their pleiotropic nature, meaning that they can exert multiple effects and function in diverse ways within different plant cells. Key phytohormones—auxin, abscisic acid (ABA), cytokinins (CKs), jasmonates (JAs), ethylene (ET), strigolactones (SLs), and gibberellins (GAs)—coordinate all aspects of plant growth and development, ensuring the proper progression through each phase of the ontogenetic cycle. Recent scientific research has increasingly shown that these compounds also exhibit biological activity in animals. Although they may exert certain adverse effects, their health-promoting properties are widely recognized and highly regarded. This has prompted extensive testing of these compounds in both in vitro and in vivo studies. They have been explored as inhibitors of tumor cell proliferation (auxins, JAs, and some GAs), inducers of apoptosis (CKs, JAs, and SLs), cosmeceuticals with antiaging agents (CKs and JAs), and potential agents for combating neurodegenerative disorders (ABA and JAs). These findings suggest possible applications as pharmacologically active compounds for addressing health disorders associated with an aging society. Extensive research today is focused on developing therapies that selectively target dysfunctional cells while preserving healthy ones. Secondary plant metabolites, a diverse class of unique molecules produced by specific plant lineages, have long been a cornerstone of conventional medicine. Increasing attention is now being directed toward phytohormones, which exhibit a broad spectrum of biological activities even at low concentrations. Emerging evidence suggests that some phytohormones may also be present in animal tissues, although their roles in these contexts remain largely unexplored. To date, exogenous phytohormones have been shown to influence various animal cells by regulating the cell cycle, inhibiting proliferation and growth, and inducing apoptosis. These findings position phytohormones as a compelling group of plant-derived compounds with a perspective for future therapeutic applications.

This paper provides an overview of recent scientific discoveries highlighting the physiological and pharmacological activities of phytohormones in animal cells. We focus on their health-promoting mechanisms while also emphasizing reported side effects that may contribute to toxicity or other health disorders in animals. Our analysis identifies specific groups of phytohormones that may function as either allies or adversaries to animal health and proposes potential directions for future research.

## 2. Phytohormones Action

### 2.1. Auxin-Related Approaches for Cancer Therapies

**Figure 1:**
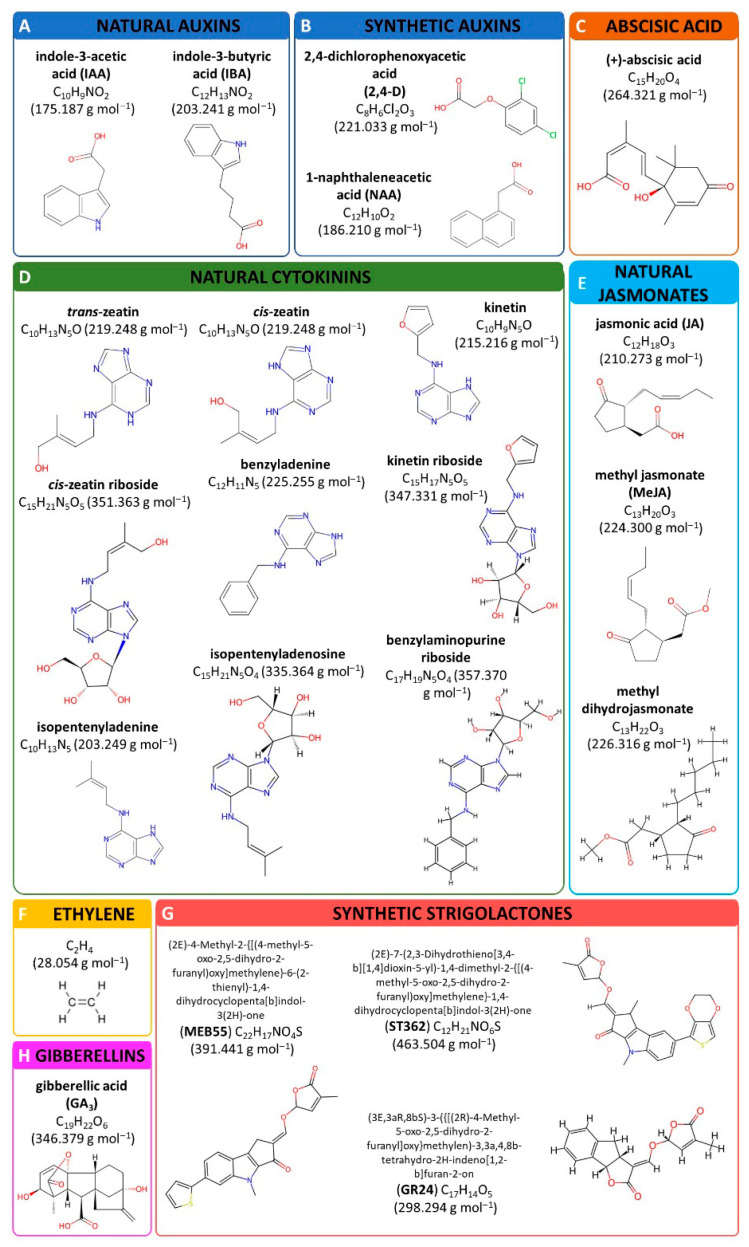
The chemical structures of the selected natural and synthetic phytohormones active in animal tissues. The structures were obtained from www.chemspider.com. For more details, see the text.

Auxins are a heterogeneous group of plant hormones primarily synthesized in young, developing tissues via either tryptophan-dependent or tryptophan-independent pathways. These hormones regulate plant cell elongation, differentiation, phototropism, and gravitropism. Auxin action in target cells requires specific receptors, Transport Inhibitor Response1/Auxin Signaling F-Box Proteins (TIR1/AFB), which, upon hormone binding, trigger the degradation of repressor proteins, including those in the Auxin/Indoleacetic Acid (Aux/IAA) family. This degradation modulates the expression of auxin-responsive genes [1]. The level of indole-3-acetic acid (IAA) (Figure 1A), the principal auxin in plant cells, is regulated by two primary metabolic pathways. The first involves the oxidation of IAA, catalyzed by peroxidases, leading to decarboxylation and subsequent degradation. The second pathway entails the formation of IAA conjugates, which expand the pool of inactive auxin forms within the plant [2].

Early studies by Weissbach et al. [3] and Gordon et al. [4] demonstrated that IAA is produced in germ-free animals and detected across multiple tissues, indicating that auxins in mammals originate endogenously rather than solely from microbial sources. Subsequent research has shown that IAA can arise in animal tissues both through microbial metabolism of dietary tryptophan and via endogenous mammalian enzymes. In the gut, bacteria convert tryptophan to indole-3-pyruvate via aminotransferases, then to indole-3-acetaldehyde by decarboxylases, and finally to IAA via aldehyde dehydrogenases [5]. IAA, as a significant microbiota-derived metabolite in humans, can be used to monitor toxin levels, e.g., following disease. Clinical trial NCT04768309 specifically investigated how variations in gut microbiota composition influence the levels of these gut-derived metabolites, which can accumulate in kidney disease and contribute to complications. These findings underscore the importance of IAA as a non-invasive marker for assessing toxin accumulation and complications associated with metabolic and renal disorders.

Mammalian immune cells express Interleukin-4-induced-1, a secreted L-amino acid oxidase that deaminates tryptophan to indole-3-pyruvate, which is subsequently converted to IAA and indole-3-aldehyde in host cells [6]. IAA activates the aryl hydrocarbon receptor (AhR), triggering receptor translocation to the nucleus and dimerization with AhR nuclear translocator (ARNT). This complex binds xenobiotic response elements to regulate gene expression, as well as influence proliferation, apoptosis, and immune responses [7,8]. IAA has also been shown to promote proliferation in mammalian renal epithelial cells [9].

In humans, 5-hydroxyindoleacetic acid (5-HIAA)—a derivative of IAA—is the primary metabolite of serotonin and has diagnostic relevance in clinical settings. The clinical trial NCT04527263 demonstrated that incorporating urinary 5-HIAA measurement into standard diagnostic protocols enhances diagnostic accuracy enabled less invasive assessment and supported improved clinical management of pediatric acute appendicitis [10].

Given the importance of maintaining auxin homeostasis and the physiological impacts of these compounds in animal systems, auxins are increasingly recognized as promising candidates for preventive healthcare applications. Moreover, both natural auxins, such as IAA and indolyl-3-butyric acid (IBA) (Figure 1A), as well as synthetic auxins like 2,4-dichlorophenoxyacetic acid (2,4-D) and naphthaleneacetic acid (NAA) (Figure 1B), have been shown to disrupt the cancer cell cycle and inhibit the proliferation of various tumor cell lines, including MCF-7 (breast cancer), SW620 and HCT116 (colon cancer), MOLT-4 (acute lymphoblastic leukemia), and H460 (lung cancer). The antiproliferative effects evoked by auxin are related to the induction of G1 phase cell-cycle arrest in human tumor cells. Auxin treatment leads to the accumulation of cells in the G1 phase, which is accompanied by a significant reduction in the number of cells entering the S phase. This blockade is associated with a marked downregulation of cyclin D1 and cyclin E expression, as well as decreased activity of cyclin-dependent kinases (CDK2 and CDK4), which are essential for G1/S phase transition [11].

Many studies on auxins in mammals focus on their enzymatic activation by horseradish peroxidase (HRP), a hemoprotein capable of oxidizing a wide range of substrates, including IAA, in the presence of H_2_O_2_ [12]. The HRP/IAA complex has attracted significant interest due to its anticancer properties in vitro [13]. Notably, neither IAA nor HRP alone exhibits cytotoxic effects. Although the precise mechanism of the HRP/IAA complex remains unclear, it is known to generate 3-methylene-2-oxidole, which binds DNA and thiol groups. Moreover, IAA oxidation produces toxic metabolites that increase reactive oxygen species (ROS), induce lipid peroxidation, disrupt the cell cycle, and activate apoptotic pathways [14,15,16,17]. The combined application of IAA and HRP reduced the viability of G361 human melanoma cells [18,19]. Similar apoptotic effects occur in these cells and in prostate cancer cells when IAA is used with ultraviolet light (UVB) treatment [20,21], suggesting that auxin may serve as a promising enhancer of photodynamic therapy.

Signaling pathways that induce apoptosis represent promising targets for anticancer therapies [22]. The human body can tolerate relatively high concentrations of IAA because endogenous mammalian peroxidases do not easily oxidize it. This selectivity permits the targeted introduction of HRP into tumor cells, where it catalyzes the formation of a toxic IAA/HRP complex that disrupts tumor function while sparing healthy tissues. To date, three approaches have been proposed to exploit this mechanism. The first is Antibody-Directed Enzyme Prodrug Therapy (ADEPT), in which a monoclonal antibody–HRP conjugate is delivered to cancer cells and activates IAA as a prodrug. The second is Polymer-Directed Enzyme Prodrug Therapy (PDEPT), which leverages the “leaky” blood vessels of tumors to allow large, polymer–HRP complexes to enter tumor tissue and locally activate IAA. The third approach is Gene-Directed Enzyme Prodrug Therapy (GDEPT), wherein HRP-encoding genes are introduced into cancer cells, leading to intracellular peroxidase expression and prodrug oxidation [23]. The cytotoxic effect of HRP-activated IAA has been demonstrated in vitro in human G361 melanoma cells [18] and TCCSUP urinary bladder carcinoma cells [24], as well as in vivo using human nasopharyngeal squamous carcinoma cells (FaDu) [25]. Despite encouraging preclinical results, none of these strategies has yet progressed to clinical trials. Major challenges include the high cost and complexity of producing and purifying HRP.

Auxins have also been shown to exert toxic effects in animals. For example, treatment of rat neutrophils and lymphocytes with IAA destabilized cell membrane integrity, induced chromatin condensation, and caused DNA fragmentation. The cytotoxicity was linked to disrupted redox homeostasis and the induction of programmed cell death [26]. Similarly, the synthetic auxin 2,4-D exhibited cytotoxic and mutagenic effects in hamster fibroblasts [27].

Interestingly, research on the digestive system has demonstrated a correlation between auxin levels and cancer progression. Specifically, the differentiation of esophageal squamous cell carcinoma was found to correlate with elevated IAA content compared to adjacent normal tissues [28]. Yamaki et al. [29] reported similar findings across various human cancers, including those of the stomach, esophagus, jejunum, colon, rectum, and breast, showing the highest IAA concentrations in tumor lesions, intermediate levels in surrounding tissue, and the lowest levels in healthy tissues, where IAA concentrations were up to ten times lower than in cancerous regions. The authors suggest that quantifying endogenous IAA in tissues could serve as a tool for early and specific cancer diagnosis.

### 2.2. Abscisic Acid as a Therapeutic Agent

Abscisic acid (ABA, Figure 1C) is a terpene hydrocarbon with diverse functions in plant growth and development, regulating processes from seed germination and flowering to senescence. ABA also mediates plant responses to environmental stress, enhancing stress tolerance [30]. Beyond plants, ABA occurs naturally in the tissues of many animals, including simple metazoans. Some phytopatogenic fungi, such as *Botrytis cinerea*, synthesize ABA via a non-plant carotenoid pathway [31]. Likewise, soil- and plant-associated bacteria (e.g., *Pseudomonas* and *Bacillus* spp.) produce ABA, influencing plant–microbe interactions and stress resilience [32]. In the sponge *Axinella polypoides*, ABA positively regulates responses to temperature fluctuations [33]. In the cnidarian *Eudendrium racemosum*, light exposure boosts ABA synthesis and accumulation; exogenous ABA increases ADP-ribosyl cyclase activity, raising intracellular Ca^2+^ levels. This enhancement improves oxygen utilization, protein synthesis, and the function of the animal’s water-filtering system under dark conditions, thereby accelerating regeneration [34].

In vitro studies of human and mouse tissues suggest that specific environmental stimuli can induce ABA production in mammals, including in granulocytes [35], pancreatic beta cells [36], and fibroblasts [37]. This observation supports the hypothesis that ABA may serve a similar function in response to abiotic factors in both primitive animals and mammals, as it does in plants. Notably, ABA synthesis pathways differ between plants and animals, as confirmed by studies on the plant-specific mevalonate pathway [38], suggesting that while ABA’s physiological effects are evolutionarily conserved, its biosynthetic routes are not. In mammals, ABA can be synthesized via carotenoid metabolism or absorbed through the digestive system. Interestingly, plasma ABA concentrations were found to increase physiologically in healthy individuals after oral or intravenous glucose administration [36]. In this context, ABA is released from the *β*-pancreatic cells and stimulates insulin secretion via cAMP signaling. Furthermore, ABA can bind to and activate Peroxisome Proliferator-Activated Receptor gamma (PPARγ), a nuclear receptor that regulates genes involved in insulin sensitivity, adipogenesis, and glucose uptake. The activation of PPARγ by phytohormone improves glucose homeostasis, enhances insulin sensitivity, and increases glucose uptake in target cells [39]. Given ABA’s potential role in glycemic regulation, further research into its function in mammals could yield promising strategies for managing lifestyle diseases such as diabetes and insulin resistance. Interestingly, the phase 2 clinical trial NCT04722354 investigated the potential beneficial effects of ABA on glucose metabolism and insulin sensitivity in adults diagnosed with prediabetes.

Mammalian response to environmental stimuli involves granulocytes, which are the first line of defense. These cells produce ROS and perform phagocytosis to neutralize pathogens. Bruzzone et al. [35] proposed that ABA may mediate this response as an endogenous cytokine regulating human granulocyte function. This regulation involves G protein activation, protein kinase A, and phospholipase C, as well as phosphorylation of ADP-ribosyl cyclase. The resulting cascade leads to overproduction of cyclic ADP-ribose and increased intracellular Ca^2+^ concentration.

A growing body of scientific evidence supports the potential of ABA as a therapeutic agent for treating various diseases. ABA’s primary receptor is the membrane protein Lanthionine synthetase C-Like2 (LanCL2), which is encoded by a gene expressed in the epithelium of immune cells [40]. LanCL2 is considered a therapeutic target in chronic inflammatory diseases and conditions with metabolic and immunological origins [41]. It is particularly significant in cardiorenal syndrome type 3 (CRS), which is characterized by acute deterioration of kidney function leading to heart failure, cardiac hypertrophy, ischemia, and arrhythmia. In rat models, the onset of these symptoms correlates with increased expression of heart-specific *LanCL2* genes. ABA positively affects cardiac function and acts as a negative regulator of transcriptional activity of disease-related genes, encoding NADPH oxidase 4 (NOX-4) and the p53 protein, both of which play significant roles in the development and progression of cardiac hypertrophy and arrhythmia [42]. Research by Guri et al. [43] in mice also suggests that ABA supplementation alleviates symptoms of inflammatory bowel disease (IBD) by reducing the expression of inflammatory factors and cell adhesion molecules and by modulating lymphocyte levels.

As early as 1986, it was demonstrated that ABA is present at higher concentrations in the brains of rats and pigs compared to other tissues, supporting the hypothesis that ABA may play a role in regulating central nervous system function [44]. Its distribution in the brain is tissue-specific; in rats, higher levels are found in the hypothalamus than in the cortex and hippocampus [45]. Given the structural similarity to retinoic acid, an increase in which is associated with depression, ABA has been investigated for its role in stress responses, particularly with the hypothalamic–pituitary–adrenal axis. This axis is regulated by corticotropin-releasing hormone (CRH), produced in the pituitary gland, which controls corticosteroid synthesis by the adrenal cortex. In rat models, severe stress triggers an increase in plasma ABA levels while its concentration in the hypothalamus decreases, coinciding with elevated blood corticosterone. These findings suggest that ABA may influence depression pathogenesis, potentially leading to new therapeutic approaches. Indeed, exogenous ABA has been shown to alleviate symptoms of chronic mild stress in rats [45,46], with its antidepressant effects attributed to the downregulation of CRH and interaction with the retinoic acid signaling pathway.

ABA has also been investigated for its potential against neurodegenerative disorders, such as Alzheimer’s disease. In transgenic mice, ABA administration was found to prevent the accumulation of pro-inflammatory markers in the nervous system and mitigate memory impairment [47]. In these conditions, inflammation and cognitive deficits often co-occur, exacerbating disease progression, and impaired insulin uptake by brain cells accelerates this process. Maze tests in rats demonstrate that ABA exerts an anxiolytic effect and enhances learning [48], which are effects attributed to its ability to cross the blood–brain barrier and modulate key signaling pathways involved in cognitive function, including protein kinase C and phosphoinositide-3-dependent kinase pathways [49,50].

Several studies have explored the effects of ABA on human mesenchymal stem cells. ABA, together with cyclic ADP-ribose, which mediates the hormone’s effects and has been found to initiate the proliferation of purified mesenchymal stem cells in vitro [51]. Additionally, ABA stimulates physiological activities of these cells, including cytokine release and prostaglandin E2 production. These findings suggest that ABA acts as an autocrine regulator of the functioning of mesenchymal stem cells and participates in paracrine signaling within tissues. Such properties could be advantageous for tissue-reconstruction research, including spinal cord repair and other conditions affecting bodily functions. Given its broad and versatile effects in mammals, ABA holds promise for modern medicinal applications, potentially leading to new drug discoveries and therapeutic strategies.

### 2.3. Promising Effects of Cytokinins

Cytokinins (CKs) constitute a diverse group of phytohormones that play key roles in numerous physiological processes in plants, including cell division, root and shoot growth, and stress tolerance. Structurally, they are adenine derivatives with either an aryl group or an alkyl chain attached to the amino group (Figure 1D). Importantly, CKs function as signaling molecules across diverse organisms in all kingdoms of life. In plants, there are two distinct CKs biosynthesis pathways. The first is plastidial de novo synthesis, catalyzed by adenylate isopentenyltransferases (IPTs), which transfer an isopentenyl group from dimethylallyl diphosphate to ATP and ADP, producing active CKs such as N6-isopentenyladenine. In the second pathway, CKs arise as by-products of tRNA modification and subsequent degradation, primarily yielding *cis*-zeatin-type CKs [52,53,54]. Phytophagous insects lack de novo *IPT* genes and instead rely on a highly active tRNA-IPT pathway, producing exceptionally high levels of N6-isopentenyladenine and trans zeatin that often exceed those found in plants. These nucleotide and riboside precursors reflect efficient tRNA modification and turnover, suggesting that insects may exploit CKs to manipulate host-plant nutrient allocation and defense responses during herbivory [55]. The biotrophic fungus *Claviceps purpurea* possesses a unique bifunctional IPT-LOG enzyme that enables de novo CKs synthesis [56]. The plant-parasitic nematode *Heterodera schachtii* secretes a functional tRNA-IPT to induce host cell division and syncytium formation [57]. In mammals, no genes encode de novo IPTs. Instead, the tRNA-bound dimethylallyltransferase (TRIT1) mediates tRNA isopentenylation in both cytosolic and mitochondrial compartments, generating N6-isopentenyladenosine modifications that, upon tRNA turnover, yield free isopentenyl derivatives. One downstream enzyme, CDK5RAP1—a radical SAM homologue of bacterial MiaB—specifically methylthiolates mitochondrial tRNAs to produce methylthiolated CKs species. CDK5RAP1 also binds cyclin-dependent protein kinase 5 (CDK5) and prevents its activation by impeding CDK5/p53 complex formation; the loss of CDK5RAP1 leads to CDK5 hyperactivity and has been linked to neurodegenerative disorders, including Alzheimer’s disease [58].

Human HeLa cells metabolize six principal CK forms: N6-isopentenyladenine, its riboside and phosphate, and the methylthio derivatives (2-methylthio-zeatin, 2-methylthio-N6-isopentenyladenine, and 2-methylthio-N6-isopentenyladenosine). CK nucleotides accumulate intracellularly, whereas free bases and ribosides are exported, indicating active interconversion and transport processes in mammalian cells [59]. Similarly, *Canis familiaris* tissues exhibit diverse CK profiles—bases, ribosides, and nucleotides—across organs such as thyroid and adrenal glands, demonstrating conserved tRNA-derived CK metabolism in vertebrates [54].

While CKs’ functions in plant biology are well established, CKs also show potential as therapeutic agents for treating human and animal diseases. One such compound is isopentenyladenosine, which has demonstrated both antiproliferative and proapoptotic effects in a wide range of tumors in vitro and in vivo studies. For instance, it inhibits proliferation [60], reduces cell viability in a time- and dose-dependent manner, and promotes necroptosis—a regulated, caspase-independent death program—in glioblastoma cell lines [61]. However, isopentenyladenosine is unstable in plasma and has a short half-life [62]. It is important to note that studies in cell and animal models have produced divergent and often ambiguous results. For example, isopentenyladenine, kinetin, and 6-benzylaminopurine (BAP) have been shown to stimulate differentiation of the HL-60 leukemia cell line into granulocytes, whereas cytokinin ribosides, such as isopentenyladenosine, kinetin riboside, and benzylaminopurine riboside, induce apoptosis. Consequently, treatments using compounds like isopentenyladenine may cause fewer side effects compared to therapies that rely on inducing cancer cell death [63]. Some diseases, including cancer, may benefit from combination therapies using multiple phytohormones. In malignant cervical cancer, the simultaneous application of a CK (ortho-methoxytopolin-riboside, MeoTR) and IAA has been found to inhibit HeLa cells’ proliferation in vitro. Notably, auxin alone does exhibit cytotoxic effects, but IAA enhances MeoTR’s inhibitory action by arresting the cell cycle in HeLa cells [64].

Several CKs have been investigated in the context of neurological diseases. Kinetin-3-glucoside, *cis*-zeatin riboside, and isopentenyladenosine counteract salsolinol-evoked toxicity—a Parkinson’s disease-like insult—in neuronal cell lines. Additionally, trans-/*cis*-zeatin, along with kinetin, reduce glutamate-induced cell death in SH-SY5Y neuroblastoma cells [65]. All analyzed CKs have been found to exhibit similarly high superoxide-radical-scavenging activity, supporting their role in protecting cells by reducing oxidative stress.

CKs also play a crucial role in human keratinocyte growth and differentiation. In vitro experiments with kinetin and Ca^2+^ ions showed increased levels of keratin 10 and involucrin, which are key markers of keratinization [66]. These findings suggest that kinetin may promote keratinocyte differentiation and have potential applications in treating conditions such as psoriasis.

Although the molecular mechanism underlying CKs’ action in mammals remains unclear, the significant ATP consumption associated with these phytohormones’ activity [67,68] suggests a possible role in maintaining cellular energy homeostasis, which is an essential factor for mitigating aging-related damage. CKs may also influence redox metabolism. Low doses of kinetin reduce apoptosis and cell death caused by oxidative stress [69]. In human skin fibroblasts, zeatin decreases H_2_O_2_ levels, prevents cell enlargement and actin polymerization, and stimulates the removal of intracellular metabolites. Zeatin-exposed cells show improved tolerance to oxidative and ethanol stress, avoiding potentially harmful or carcinogenic effects [70]. These antiaging effects of zeatin, attributed to its antioxidant properties, offer promising avenues for both cosmetic and medical industries.

### 2.4. Jasmonates—Battle Against Cancer

Jasmonates (JAs) are a class of phytohormones that include jasmonic acid (JA) and its various derivatives and conjugates, e.g., the methyl derivative (methyl jasmonate, MeJA) or those formed with amino acids like isoleucine, valine, tryptophan, tyrosine, and phenylalanine, as presented in Figure 1E. Their levels in plant tissues decline with age but accumulate under specific conditions, including in response to biotic factors (pathogens), mechanical damage (wounding), or physical stress (UV radiation). In this context, JAs induce defense mechanisms by promoting ROS production and secondary metabolites synthesis, which together protect plants from stressors. JAs are synthesized from α-linolenic acid, which is released from chloroplast membranes in a lipase-catalyzed reaction [71]. Comparative studies show that non-plant taxa, such as fungi, bryophytes, or charophytes, possess orthologs of core JA-biosynthetic enzymes, LIPOXYGENASE (LOX), ALLENE OXIDE SYNTHASE (AOS), and CYCLASE (AOC), as well as OXOPHYTODIENOATE REDUCTASE (OPR), that differ in subcellular localization, substrate specificity, and downstream modifications [72,73,74,75,76]. JAs, their precursors or conjugates, occur in fungi, *Bryophytes*, and *Charophytes* [76,77]. In turn, animals lack the plastid-localized enzymes of the canonical octadecanoid pathway and therefore cannot endogenously synthesize JAs via this route [78]. Nevertheless, JAs are fatty acid derivatives structurally similar to animal prostaglandins—hormones noted for their anti-inflammatory properties [79].

Experimental studies have demonstrated that JAs can influence animal physiological activity, exhibiting cytotoxic effects on cancer cells without impairing normal cells [80]. This JAs-dependent response was first documented in 2002 in studies on human lymphoblastic leukemia cells. Both free JA and MeJA have been shown to induce apoptosis through changes in nuclear morphology and caspase activation [81]. Given the anticancer potential of JAs, particular attention has been focused on MeJA, which exhibits greater cytotoxicity toward cancer cells compared to free JA. MeJA has been found to inhibit the proliferation of various cancer cell lines, including mouse and human breast, as well as colon, prostate, and lymphoma cells [82,83]. Zhang et al. [84] have shown that MeJA induces apoptosis in human non-small cell lung cancer cells via the DDIT3–TNFRSF10B–caspase axis, where the stress-induced transcription factor DDIT3 upregulates the death receptor (TNFRSF10B), leading to caspase activation. Moreover, MeJA triggers pro-apoptotic autophagy through the generation of ROS, critical mediators of both apoptosis and autophagy, highlighting MeJA’s potential as a selective anticancer agent targeting multiple cell death pathways.

One JAs-dependent pathway involved in mediating cell death is associated with mitogen-activated protein kinases (MAPKs). MeJA has been shown to influence MAPK signaling in acute lymphoblastic leukemia [85] and lung cancer cells [86]. However, the most well-characterized mechanism induced by MeJA to date involves mitochondrial dysfunction. Studies on chronic lymphocytic leukemia cells and isolated mitochondria have demonstrated that MeJA triggers membrane depolarization, swelling of the organelles (linked to the disruption of osmotic balance between the mitochondrial matrix and cytosol), and the release of cytochrome c. These effects are related to dysfunction of the permeability transition pore complex (PTPC), which forms channels at the contact site between the inner and outer mitochondrial membranes. Prolonged opening of these channels leads to the release of mitochondrial content, contributing to cell death. Importantly, mitochondria isolated from lymphocytes of healthy tissues did not exhibit similar changes when exposed to exogenous MeJA [87].

This hormone has also been shown to modulate glycolysis in cancer cells. The first enzyme in this pathway, hexokinase II, catalyzes glucose phosphorylation. In tumor cells, hexokinase II levels are over 200 times higher than in healthy tissues, making it a promising target for drugs used in targeted therapy [88]. The elevated glucose phosphorylation catalyzed by hexokinase II supports the rapid growth rate and viability of cancer cells [89]. MeJA binds to and detaches this enzyme from the mitochondrial membrane, thereby reducing glycolysis by decreasing the availability of active glucose molecules. This results in the loss of mitochondrial membrane potential, cytochrome c release, rapid ATP depletion, and, ultimately, apoptosis. The anticancer activity of MeJA has been demonstrated in several human cancer cell lines, including melanoma, colon cancer, and leukemia in mice, as well as acute lymphoblastic leukemia [90]. In mouse models of multiple myeloma, treatment with exogenous MeJA extended the reported lifespans [91]. The selective anticancer effect of MeJA is thought to arise from its inhibition of hexokinase, which is more detrimental to cancer cells compared to normal cells, as the former relies heavily on glycolysis for energy production [92].

Particularly promising are the findings from research on the effects of synthetic JA derivatives on glioma, the most common and aggressive brain tumor in adults. These compounds act by inhibiting the activity of hexokinase II, which, as previously mentioned, plays a critical role in cancer progression. A newly synthesized MeJA analog (C-10), which functions as a hexokinase II inhibitor, induces autophagy, necrosis, and apoptosis in glioma cells more effectively than MeJA alone [93]. Similarly, the C3 derivative has demonstrated comparable anticancer properties in studies on lung cancer [94]. Thus, developing JA derivatives with enhanced anticancer activity represents a key challenge for modern medicine.

MeJA also exhibits anti-inflammatory effects, as demonstrated in studies on rats with arthritis. These conditions are often accompanied by disruption in cellular redox balance in cells, as observed in the liver of arthritic rats. The health-promoting properties of MeJA in this context are attributed to two key mechanisms: reducing inflammation and stimulating the endogenous antioxidant system. In the livers of diseased rats, MeJA treatment resulted in the decreased activity of myeloperoxidase (MPO), an enzyme involved in inflammatory responses, and increased catalase (CAT) activity, along with an elevated ratio of reduced glutathione (GSH) to its oxidized form. Additionally, MeJA enhances the function of the ROS inactivation system by modulating the microRNA expression. Specifically, it decreases the expression of *miR-155* and increases that of *miR-101*. As a consequence, the activity of Nfr2, a transcription factor that upregulates genes encoding CAT and enzymes essential for the synthesis and regeneration of GSH, is enhanced [79]. Moreover, MeJA has been shown to downregulate the expression of genes encoding pro-inflammatory enzymes and cytokines. In the brains of mice with neuroinflammation, MeJA suppresses the production of pro-inflammatory mediators such as nitric oxide, prostaglandin E, tumor necrosis factor (TNF-α), interleukins 1 and 6 (IL-1 and IL-6), as well as the expression of genes encoding inducible nitric oxide synthase (*iNOS*) and cyclooxygenase [95,96].

The anti-inflammatory and anti-amnesic effects of MeJA show great potential in combating neurodegenerative diseases, such as Alzheimer’s disease. This hormone has been shown to reverse memory disorders in mice, as evidenced by reductions in biomarkers of neuroinflammation, including prostaglandins, interleukins, cyclooxygenases, and iNOS [96]. Notably, several synthetic JA derivatives were found to exhibit even stronger activity than naturally occurring anti-inflammatory prostaglandins [97]. In murine macrophage cells treated with lipopolysaccharide to induce inflammation, synthetic JAs (methyl dihydrojasmonate, J2) have been found to reverse these inflammatory effects, resulting in reduced secretion of pro-inflammatory cytokines, particularly TNF-α and IL-6 [98].

JA derivatives are gaining attention in contemporary research not only for their therapeutic potential but also for their promising applications in cosmetology. One notable example is tetrahydrojasmonic acid (commercially known as LR2412), which has been shown to stimulate collagen production, exfoliate the skin, and reduce signs of aging [99]. Moreover, LR2412 enhances the formation of hyaluronic acid in the skin, contributing to increased skin thickness [100]. Aesthetic medicine also holds promise for a newly synthesized JA conjugate (JA–YPFF–NH_2_), which combines JA with the tetrapeptide YPFF (Tyr-Pro-Phe-Phe-NH_2_), a peptide widely used in cosmetology [101]. The peptide alone has been found to diminish the stimulation of nerve endings, thereby reducing skin hypersensitivity [102].

### 2.5. Ethylene—An Animal Stress Hormone

Ethylene (ET), synthesized from methionine via 1-aminocyclopropane-1-carboxylate synthase and oxidase (ACC and ACO), is a simple molecule (Figure 1F) that coordinates numerous processes in plant growth and development. In the 1920s, ET was used in medicine as an anesthetic for minor surgical procedures and operations [103]. Its administration was straightforward, and patients did not experience common side effects such as nausea. ET did not cause acidosis, respiratory irritation, or affect the nervous system. However, due to its high flammability and the potential for physiological effects and unpleasant odor at high doses, ET has since been replaced by more modern anesthetic agents [104]. This gaseous phytohormone, often referred to as the “stress hormone,” is produced in large amounts by plants during aging and in response to abiotic stresses, such as wounding, and biotic stresses, such as pathogen infections [105]. However, ET is not exclusive to plants; it is also produced by bacteria, fungi, algae, and bryophytes through distinct biosynthetic routes. Most bacteria and fungi generate ET via 2-oxoglutarate-dependent oxygenases (EFEs) or through non-enzymatic transformation of 2-keto-4-methylthiobutyric acid (KMBA) to ET, for example, by photo-oxidation [106,107]. These alternative pathways highlight the existence of multiple evolutionary solutions to ET biosynthesis outside of the plant pathways. Photosynthetic eukaryotes such as algae and *Bryophytes* possess homologs of plant ACS and ACO and demonstrate ACC-dependent ET production [108]. While no animal homologs of the enzymes responsible for ET biosynthesis have been identified, the presence of ET in animals has been confirmed. As early as the 1960s, studies showed that administering copper and nickel ions to rats led to ET accumulation in liver microsomes [109,110]. A similar effect was observed in mice following exposure to carbon tetrachloride [111]. ET can be a final product of reactions between free radicals and polyunsaturated fatty acids, making its measurement a potential indicator of plasma membrane damage. However, ET is not solely a secondary product of lipid peroxidation; it can also be produced from the oxidation of methionine [112] or hemoglobin [113].

Scientific reports have demonstrated the production of ET in human tissues, such as skin exposed to UV radiation [114]. Recent research has revealed that ET is released in the exhaled air of patients and produced as a result of systemic inflammation and fatty acids breakdown [115]. This suggests that ET could serve as a biological marker of physiological disorders. In cardiac surgery, this property of ET is particularly valuable: the hormone’s concentration rises from the early stages of the procedure and remains elevated throughout [116], allowing for real-time, non-invasive monitoring of a patient’s oxidative stress without the need for multiple sample collections and analyses.

The application of ET alters metabolism in simpler organisms such as sea sponges (*Suberites domuncula*) [117]. Exposure to this phytohormone increases intracellular Ca^2+^ concentration, which is crucial for cell signaling and helps inhibit apoptosis caused by nutrient deficiency. A similar signaling role of ET is likely present in more complex organisms. The treatment of mouse and human cell lines with ethephon (a donor of ET in living tissues) resulted in a rapid, concentration-dependent accumulation of Ca^2+^ ions and an increase in cell division rates [118]. Furthermore, ET generation occurs before the appearance of pro- and anti-inflammatory cytokines in humans, making it an early and effective biomarker for oxidative stress during infection [115]. However, the molecular mechanisms underlying mammalian cellular responses to ET remain unclear. Unlike other phytohormones, ET has not been extensively studied in the context of health-related issues, highlighting critical gaps that require further investigation.

### 2.6. Strigolactones—Antiproliferative Agents

Strigolactones (SLs, Figure 1G)—phytohormones derived from carotenoids—are native to land plants (including bryophytes and charophyte algae) but are not endogenously produced by fungi, bacteria, or chlorophyte algae [119]. Animals also lack orthologs of the core SL biosynthetic enzymes. SLs regulate various plant processes, including shoot and root branching, aging, and stress responses [120]. Recent research has expanded the significance of SLs beyond plants, revealing their potential roles in animals, where they exhibited anticancer and anti-inflammatory effects. Initial studies published in 2012 demonstrated that synthetic SLs analogs reduced mammosphere growth and inhibited the proliferation of breast cancer cells. This inhibition was associated with cell cycle arrest in the G2/M phase and the induction of apoptosis [121]. The cytotoxic effects of these analogs were notably more pronounced in cancer cells compared to normal cells. At the cellular level, synthetic SLs activated a stress response mediated by MAP kinases (P38 and JUN N-TERMINAL KINASES1/2-JNK1). Under this condition, the phosphorylation of p38 activates the p53 protein, which subsequently leads to cell death [121]. Disruption of the cell cycle and induction of apoptosis were also observed with synthetic SLs (IND and EGO10) in the glioblastoma multiforme [122]. Another group of chemically synthesized SLs analogs (MEB55 and ST362, Figure 1G) has been shown to inhibit the growth of various cancer cell types, including prostate, colon, lung, melanoma, osteosarcoma, and leukemia. Similar to their effects in breast cancer, these compounds cause the phosphorylation of p38 and JNK1/2 kinases and activate target proteins, including heat shock proteins (HSPs), such as HSP27 and HSP70 [123]. MEB55 and ST36 were also found to induce double-strand DNA breaks—one of the most severe and mutagenic forms of DNA damage—and to inhibit DNA repair processes in osteosarcoma cells [124].

Detailed analyses aimed at elucidating the action of SLs have revealed that their effects on diseased cells are comparable to those of paclitaxel—a well-known chemotherapeutic drug. In response to exogenous SLs (MEB55), breast cancer cell lines (MDA-MB-231) exhibited altered microtubule integration compared to untreated cells. This modification in microtubule positioning is crucial for cell migration, a process that is closely associated with metastases formation. Moreover, an additive effect of synthetic SLs in combination with paclitaxel has been observed, enhancing the effectiveness of these treatments and resulting in greater inhibition of cancer cell growth [125].

Furthermore, a synthetic SL (GR24) has been shown to inhibit the release of pro-inflammatory mediators, such as NO, TNf-α, and IL-6 in *Danio rerio* [126]. The anti-inflammatory activity of GR24 has also been demonstrated in human microglial cancer cell lines [127], supporting the rationale for in vivo studies and its potential use in combating neurodegenerative disorders and the early stages of Alzheimer’s disease. Additionally, GR24 influences cellular energetics by regulating glucose metabolism in rat skeletal muscle, enhancing the activity of NAD^+^-dependent deacetylase-sirtuin (SIRT1)—a molecule crucial for maintaining glucose homeostasis and modulating insulin levels [128]. Collectively, SLs show strong therapeutic potential beyond their roles in plants; however, further in vivo research is needed to fully assess their clinical promise.

### 2.7. Dual Face of Gibberellins

Gibberellins (GAs) are a group of diterpenoid plant hormones consisting of over 130 compounds, although only a subset exhibits physiological activity (Figure 1H). These phytohormones play a crucial role in regulating seed germination, hypocotyl elongation, flower development, and fertilization. In plants, the GID1 (GIBBERELLIN INSENSITIVE DWARF1) receptor binds GA and interacts with DELLA repressor proteins, leading to their degradation via the proteasome, which is a process mediated by the SCF^GID^ (SKP1-CULLIN-F-BOX complex with the F-box protein subunit GID) ubiquitin ligase. The removal of DELLA proteins enables the activation of GA-responsive genes [129].

The principal representative of GAs, gibberellic acid (GA_3,_ Figure 1H), also influences various biological parameters in animals. In rats, GA_3_ reduces the blood levels of glucose, urea, creatinine, and malondialdehyde—a marker of lipid peroxidation [130,131]. Conversely, exogenous GA_3_ negatively affects sperm count and genetic material quality, leading to reduced fertility in this species [132]. Similarly, GA_3_ has been shown to lower testosterone levels in rabbits [133].

GAs have been shown to induce significant changes in the structure and function of internal organs. Histological analysis of kidney sections from rats exposed to GA_3_ revealed the development of interstitial fibrosis characterized by glomerulosclerosis. GA_3_ exposure was also found to lead to liver fibrosis and the formation of necrotic lesions in animals [131]. Growing evidence from studies on rats indicates that the detrimental effects of GA_3_ are linked to disruptions in the redox homeostasis. Specifically, GA_3_ exposure is associated with reduced activity of key antioxidant enzymes, such as superoxide dismutase (SOD), CAT, and glutathione reductase (GR) in the spleen, heart, liver, and kidneys [134,135,136]. Moreover, the exposure of rats to GA_3_ during late pregnancy or lactation has been shown to induce oxidative stress in their offspring and cause nephrotoxic effects.

The adverse effects of GAs extend to their potential carcinogenicity, as evidenced by studies in humans, rodents, and amphibians. For example, administering GA_3_ to toads (*Bufo regularis*) led to the development of liver, kidney, and ovarian cancer in 16% of the tested animals [137]. In mice, GA_3_ exposure resulted in tumors in the armpits, breasts, and lungs of 18% of males and 36% of females [138]. Additionally, GA_3_ has been shown to damage the DNA of both mouse and human lymphocytes [138,139]. Interestingly, the cytotoxic effects of GA_3_ on human lymphocytes can be mitigated by the polyphenols found in green tea. These polyphenols possess anticancer and antimutagenic properties, exerting antioxidant effects through free radical scavenging and stimulation of detoxification systems [140]. Given the high toxicity of GAs and their potential health risks, it is important to recognize that we are continually exposed to trace amounts of GAs through ripening agents used in the production of fruits and vegetables.

For these reasons, natural GAs have not been utilized in medicine. However, derivatives of GAs have been synthesized, such as those incorporating two α- and β-unsaturated ketone units into otherwise inactive GAs, resulting in compounds with demonstrated anticancer properties. This activity has been confirmed through in vitro cytotoxicity assays, including MTT tests conducted on human colorectal (HT29), lung (A549), liver (HepG2), and stomach (MKN28) cancer cell lines. One notable derivative, GA-13315 (13-chlorine-3,15-dioxy-gibberellic acid methyl ester), was found to completely inhibit the activity of topoisomerase I, which is an enzyme that regulates DNA supercoiling [141]. Consequently, it is proposed that GA-13315 acts by disrupting the DNA structure. Further detailed analyses, both in vitro and in vivo, have demonstrated that GA-13315 significantly inhibits cell division in hematological cancer cell lines as well as in three solid-tumor cell lines, without exhibiting cytotoxic effects in normal tissues. In mouse models of lung cancer, GA-13315 displayed notable anticancer effects with minimal side effects. Additionally, GA-13315 was found to inhibit angiogenesis—the formation of new blood vessels from pre-existing ones. While angiogenesis is the normal process involved in wound healing and other physiological states, it also plays a critical role in pathological conditions such as cancer, where tumors rely on new blood vessels to supply oxygen, nutrients, and growth factors for their development. The antiangiogenic effect of GA-13315 is attributed to its ability to block the receptor for Vascular Endothelial Growth Factor (VEGF), which is a key pro-angiogenic cytokine [142]. These findings strongly suggest the need for further research on GA derivatives that can inhibit cancer cell growth without the severe adverse effects associated with GA_3_.

Clinical trials investigating GAs have primarily focused on their potential implication for human health, particularly through dietary exposure and allergenic sensitization. A notable example is clinical study NCT06183398, which examined the prevalence of sensitization to gibberellin-related proteins (GRPs) present in various fruits and vegetables, including peaches, apples, sesame, carrots, lemons, and oranges. This study employed skin prick tests to evaluate allergic responses in patients, with the goal of enhancing understanding of the risk and management strategies associated with severe food allergies linked to GRPs.

## 3. Conclusions

This review highlights how plant hormones can act as protectors, aggressors, or even toxins within animal systems. Some phytohormones are obtained from the diet, while others are produced endogenously in animal tissues. Notably, the research suggests that exogenous plant hormones and their derivatives possess potent anticancer properties, particularly against common cancers, such as those of the lung, colon, breast, liver, cervix, and stomach. There is also strong evidence indicating a possible role of these compounds in the treatment of diabetes and neurodegenerative disorders, which are diseases closely linked to an aging population. Additionally, the review underscores promising applications in cosmetology, especially for antiaging purposes. It is worth noting that the majority of phytohormone studies are still at the preclinical or basic research stage. Currently, the most prominent trial involving plant hormones focuses on their dietary intake, allergic sensitization, and diagnostic and biomarker applications, as well as therapeutic investigations.

The data reviewed strongly suggests that caution is warranted; phytohormones should not be labeled as strictly “beneficial” without sufficient experimental evidence to support their safety and efficacy.

**Figure 2:**
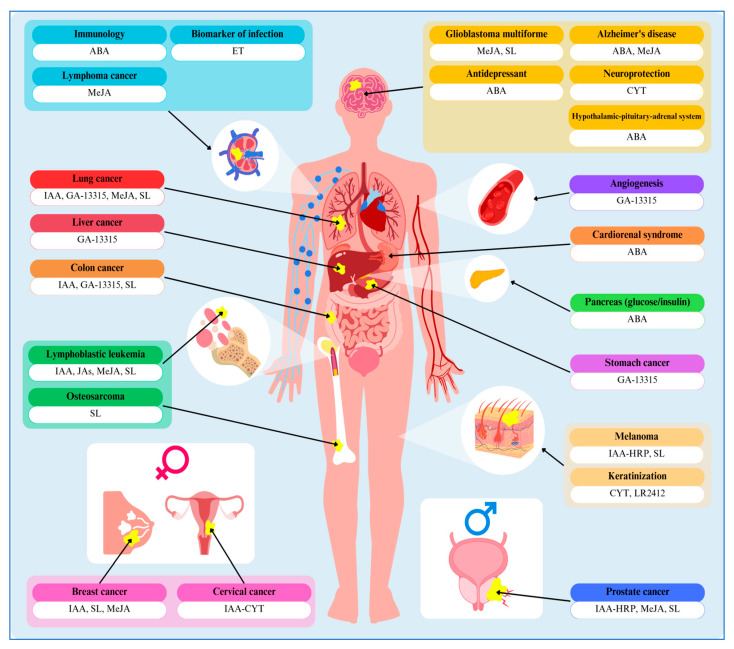
Diversity of beneficial activities of plant hormones toward human disease and disorders. Abbreviations: ABA—abscisic acid, CYT—cytokinin, ET—ethylene, GA-13315—13-chlorine-3,15-dioxy-gibberellic acid methyl ester, IAA—indole-3-acetic acid, IAA-HRP—indole-3-acetic acid/horseradish peroxidase complex, JAs—jasmonates, LR2412—tetrahydrojasmonic acid (trade name), MeJA—methyl jasmonate, SL—strigolactone.

Looking ahead, these plant-derived compounds offer exciting therapeutic potential, as illustrated in Figure 2, with particular promise in stabilizing homeostasis and advancing innovative oncology treatments. Key research focuses on exploring how plant hormones influence animal and human physiology, with the potential to uncover novel functions in animal science. As biotechnology and biomedical research advance, the possibilities of these molecules to yield new insights continue to grow. A central question remains: do plant hormones operate via the same pathways in mammalian cells as in plants, or do they engage distinct mechanisms? Resolving this question may therapeutic avenues that span plant, animal, and human biology. To fully realize the promise of phytohormones in human health, future studies should prioritize (1) elucidating the molecular mechanism by which these compounds act in animal models and, by extension, in humans; (2) developing strategies to minimize adverse or harmful effects and ensure both safety and efficacy; and (3) identifying and characterizing new phytohormones with even greater therapeutic potential.

## Data Availability

No new data were created or analyzed in this study. Data sharing is not applicable to this article.
